# Mechanisms of Action of Extracorporeal Photopheresis in the Control of Bronchiolitis Obliterans Syndrome (BOS): Involvement of Circulating miRNAs

**DOI:** 10.3390/cells11071117

**Published:** 2022-03-25

**Authors:** Sara Bozzini, Claudia Del Fante, Monica Morosini, Hatice Oya Berezhinskiy, Sophia Auner, Elena Cattaneo, Matteo Della Zoppa, Laura Pandolfi, Rosalia Cacciatore, Cesare Perotti, Konrad Hoetzenecker, Peter Jaksch, Alberto Benazzo, Federica Meloni

**Affiliations:** 1 Laboratory of Respiratory Disease, Cell Biology Section, Fondazione IRCCS Policlinico San Matteo, 27100 Pavia, Italy; m.morosini@smatteo.pv.it (M.M.); cattaneoelena90@gmail.com (E.C.); matteo.dellazoppa@gmail.com (M.D.Z.); pandolfi.la@gmail.com (L.P.); 2 Immunohaematology and Transfusion Service, Apheresis and Cell Therapy Unit, Fondazione IRCCS Policlinico San Matteo, 27100 Pavia, Italy; c.delfante@smatteo.pv.it (C.D.F.); r.cacciatore@smatteo.pv.it (R.C.); c.perotti@smatteo.pv.it (C.P.); 3 Department of Thoracic Surgery, Medical University of Vienna, 1090 Wien, Austria; hatice.akyildiz@meduniwien.ac.at (H.O.B.); n1508860@students.meduniwien.ac.at (S.A.); konrad.hoetzenecker@meduniwien.ac.at (K.H.); peter.jaksch@meduniwien.ac.at (P.J.); alberto.benazzo@meduniwien.ac.at (A.B.); 4 UOS Transplant Center, Fondazione IRCCS Policlinico San Matteo, 27100 Pavia, Italy; f.meloni@smatteo.pv.it

**Keywords:** circulating microRNAs, BOS, ECP

## Abstract

Clinical evidence suggests an improvement or stabilization of lung function in a fraction of patients with bronchiolitis obliterans syndrome (BOS) treated by extracorporeal photopheresis (ECP); however, few studies have explored the epigenetic and molecular regulation of this therapy. The aim of present study was to evaluate whether a specific set of miRNAs were significantly regulated by ECP. Total RNA was isolated from serum of patients with established BOS grade 1–2 prior to the start and after 6 months of ECP treatment. We observed a significant downregulation of circulating hsa-miR-155-5p, hsa-miR-146a-5p and hsa-miR-31-5p in BOS patients at the start of ECP when compared to healthy subjects. In responders, increased miR-155-5p and decreased miR-23b-3p expression levels at 6 months were found. SMAD4 mRNA was found to be a common target of these two miRNAs in prediction pathways analysis, and a significant downregulation was found at 6 months in PBMCs of a subgroup of ECP-treated patients. According to previous evidence, the upregulation of miR-155 might be correlated with a pro-tolerogenic modulation of the immune system. Our analysis also suggests that SMAD4 might be a possible target for miR-155-5p. Further longitudinal studies are needed to address the possible role of miR-155 and its downstream targets.

## 1. Introduction

Lung transplantation (LTx) is a therapeutic option for selected patients with end-stage lung disease [1]. However, the onset of bronchiolitis obliterans syndrome (BOS), the obstructive phenotype of chronic lung allograft dysfunction (BOS), hinders long-term survival after LTx [2].

Despite many immunosuppressive drugs and novel therapies that have been developed in the last decade to prevent immunological rejection, there are no established treatment of BOS. Extracorporeal photopheresis (ECP) has emerged as a promising treatment in patients who develop CLAD [3]. Several studies have indicated the immunomodulatory effect of ECP therapy on patients after solid organ transplantation, in particular in the setting of chronic rejection [4,5,6,7]. Few studies analyzed the ECP mechanism of action in CLAD, and thus the most broadly accepted mechanistic hypothesis is derived from studies in GVHD patients [8]. This includes an initial activation of immature dendritic cells (DCs) by an inflammatory cascade, followed by an expansion of plasmacytoid DC with a stimulation of tolerogenic mechanisms and inhibition of allospecific effectors [9]. Some of these mechanisms have also been suggested in lung recipients, but remain inconclusive. Few studies have explored the epigenetic and molecular regulation mediated by ECP, and to date, no data are available on microRNAs (miRNAs) dysregulation resulting from this treatment in BOS patients. MiRNAs are small, non-coding RNA that can inhibit gene expression at the post-transcriptional level by binding to the 3′UTR of target messenger RNAs, thereby promoting their degradation or inhibiting translation [10]. The aberrant expression of miRNAs is associated with initiation and progression of pathological processes including immune-mediated disorders, cancer and fibrosis [11,12,13]. Moreover, miRNAs are found to be involved in regulating the differentiation and proliferation of specific immune subsets [14].

The aim of the present study was to evaluate the molecular regulation underlying the effects of ECP and to analyze the differential expression of miRNAs potentially involved in ECP response in CLAD patients. Moreover, our aim was to evaluate whether the dysregulation of any miRNAs could provide insights on ECP mechanisms of action.

## 2. Materials and Methods

### 2.1. Study Design

This is a comparative pilot study, including serum samples of LTx recipients who developed BOS and started ECP, prospectively collected between 1995 and 2015. Twenty-six adult patients with BOS were treated with ECP: 16 patients subject to LTx from 1995 to 2006 at the Medical University of Wien started ECP by on-line methods, and 10 patients, transplanted from 2003 to 2015 at IRCCS Policlinico S. Matteo Foundation in Pavia, started ECP by off-line method at Apheresis Unit of Immunohaematology and Transfusion Service. Venous blood samples were collected from patients at two time-points: at ECP enrollment and after 6 months of treatment. The serum was separated by centrifugation at 2000 g for 10 min, followed by 15 min high-speed centrifugation to completely remove the cell debris. To minimize degradation, samples were processed within 2 h. Serum samples were then stored at −80 °C until further analysis. At the same time-points, peripheral blood mononuclear cells (PBMCs) and bronchoalveolar lavages (BAL) supernatants, derived from four BOS patients, were collected. Inclusion criteria were primary lung transplantation, adult age and serum samples availability both at time of ECP beginning and 6 months after ECP treatment. Exclusion criteria were multi-organ transplantation and age ≤18. The 16 control patients were healthy subjects matched for gender and age. All subjects included in the study provided written consent to the use of anonymized personal and clinical data prior to treatment (the study was approved by the ethical committee of the San Matteo Foundation n°68792/2018).

### 2.2. ECP Procedures

On-line ECP was performed using the THERAKOS^TM^ photopheresis system (Therakos (UK) Ltd., Surrey, UK, a Mallinckrodt Pharmaceuticals company), which is a closed-loop sterile system. The procedure is described in detail elsewhere [15]. During ECP, peripheral blood mononuclear cells were separated from the whole blood in a Latham centrifuge (Latham International, Chesterton, UK) at 2700 rpm. The collected cells (buffy coat bag) were treated with 8-methoxypsoralen solution (Uvadex; Therakos) and exposed extracorporeally to ultraviolet A light (1–2 J/m^2^) before reinfusion into the patient. During each treatment, 4–6 collection cycles were performed, or 1500 mL of peripheral blood was processed, depending on the patient’s hematocrit level.

When ECP was performed using off-line technique, PBMCs were collected from the patient using a cell separator device, processing 1.5–2 blood volumes. Hemocytometric analysis was performed on the product at the end of each collection (quality control). Then, cells were irradiated (UV-A at 2 J/cmq; Macogenic, Macopharm a, France) after the dilution with saline solution and the addition of 8-methoxypsoralen (at 200 ng/mL concentration). Finally, the photoactivated PBMCs were immediately reinfused into the patient [16]. During the entire ECP procedure, vital parameters, such as blood pressure, heart rate and oxygen saturation, were monitored. Major reinfusion adverse events were defined as asthma, bronchospasm, dyspnea or bleeding [16]. Patients whose FEV1 declined less than 10% with respect to basal value were classified as responders.

### 2.3. miRNAs Selection

For the present study, we selected fourteen miRNAs previously reported as involved in regulating the proliferation and function of the main immune cell types of the innate and adaptive immunity. In particular, we investigated the relative expressions of hsa-miR-155-5p, hsa-miR-146a-5p, hsa-miR-31-5p, hsa-miR-125a-5p, hsa-miR-30b-5p, hsa-miR-99a-5p, hsa-miR-17-5p, hsa-miR-23b-3p, hsa-miR-98-5p, hsa-miR-182-5p, hsa-miR-181a-3p, hsa-miR-21-5p, hsa-miR-24-3p and hsa-miR-223-5p in serum samples. These miRNAs were reported to be involved in the regulation of immonospoppressive properties of dendritic cells (DCs), B cells ad T cells, the major cell types that play critical roles in maintaining tolerance and/or driving rejection of grafts. In particular, miR-30b, miR-125a, miR-99a, miR-17 and miR-23b were previously demonstrated to be dysregulated in tolerogenic DC [17]. Previous studies showed that miR-181 expression increased the production of B lymphoid lineage cells both in vivo and in vitro, and miR-155 was found to be required by B-cells for high-affinity antibody production and immunoglobulin class switching [18]. miR-17–92 cluster was also shown to regulate B-cell proliferation, development and immunoglobulin rearrangement [19]. In addition, some miRNAs, such as miR-125a, miR-155, miR-182, miR-181a, miR-21, miR-24, miR-31 and miR-146a, were reported to be potentially involved in T cell development and the regulation of Treg differentiation and function [20,21,22].

### 2.4. miRNAs Analysis

RNA was isolated from serum using miRNeasy serum/plasma kit (Qiagen, Germany). RNA concentration and purity were assessed using an Eppendorf Biophotometer. RNA concentrations ranged from 25 to 50 ng/μL, and the purity was verified by use of A260/A280 ratios (range 1.7–1.9). Complementary DNA (cDNA) was synthesized with miRCURY LNA RT Kit (Qiagen) at 42 °C for 60 min and 95 °C for 5 min. For the quality control of differences in RNA extraction or RT efficiencies, a synthetic cel-miR-39 was utilized as spike-in control RNA. Real-time PCR analysis was performed to evaluate miRNAs’ expression levels using miRCURY LNA miRNA PCR-specific Detection Probe and miRCURY LNA SYBR Green PCR Kit (Qiagen) with a LightCycler 480 (Roche, Switzerland), according to the manufacturer’s recommendations. Thermal cycling conditions consisted of initial denaturation at 95 °C for 10 min, followed by 45 cycles of 95 °C for 10 s followed by 60 °C for 1 min. The threshold cycle (Ct) was defined as the fraction cycle number at which fluorescence exceeded the given threshold. The stable Ct values of cel-miR-39 obtained from all spike-ins indicated successful RNA isolation, reverse transcription and qPCR detection system.

Expression levels of the small nuclear RNA RNU6 were used as the normalization control. RNU6 was chosen as candidate reference for this study on the basis of our previous experience and literature data. Serum U6 levels were analyzed by quantitative qPCR and used as reference genes based on the criteria that it was expressed in all samples. The mean Ct value < 35 and it was not significantly different among the subgroups of the population studied (*p* > 0.05).

Expression levels of dysregulated miRNAs were evaluated in BAL supernatant, as described above. BAL of four BOS patients, prior to the start and after 6 months of ECP treatment, were collected. All bronchoscopies were performed for diagnostic purposes. BALs were centrifuged at 400 rpm for 10 min at room temperature, and BAL supernatants were then stored at −20 °C until analysis.

Each experimental condition was performed in triplicate. Relative quantifications were calculated with the comparative Ct method.

### 2.5. miRNAs Target Prediction and Pathway Analysis

DIANA miRPath v.2.0 was used to predict target genes ofdysregulated miRNAs into know KEGG (Kyoto Encyclopedia of Gene and Genome) pathways [23]. The output of the program provides an overview of the parts of the pathways modulated by miRNAs. The statistical significance value associated with the identified signaling pathways and biological process was calculated by the program.

### 2.6. miRNAs and Targets Expression Analyses in PBMCs

In order to validate some of the specific targets identified by prediction analysis, we decided to concentrate further on processes of lung fibrogenesis and graft tolerance, such as STAT3, SMAD3 and SMAD4 [24,25,26,27]. RNA was isolated from PBMCs derived from 4 BOS patients, prior to the start and after 6 months of ECP treatment, by miRNeasy Mini Kit (Qiagen). MiR-155-5p and miR-23b-3p expression levels were evaluated as described above. To evaluate STAT3, SMAD3 and SMAD4 gene expression, cDNA was retro-transcribed from 1 µg of total RNA using LunaScript RT SuperMix Kit (NEB). Relative levels of STAT3, SMAD3 and SMAD4 mRNA were assessed using SYBR**^®^** Green Luna**^®^** Universal qPCR Master Mix (NEB) and normalized to the levels of glyceraldehyde-3-phosphate dehydrogenase (GAPDH) mRNA. Each experimental condition was performed in triplicate. Relative gene expression level quantification was compared with internal standards and analyzed using the 2^−ΔΔCt^ method.

### 2.7. Enzyme-Linked Immunosorbent Assay (ELISA)

To quantify TGF-β released in the BAL supernatant, ELISA assays were performed. We quantified TGF-β (Abcam) following the manufacturer’s instructions and the results were expressed as pg mL^−1^.

### 2.8. Statistical Analysis

The mean and standard deviation ore median and interquartile range are presented for continuous variables, and numbers and percentages are presented for categorical variables. Groups were compared to parametric or non-parametric tests, according to data distribution, for continuous variables. Correlations were calculated using Spearman’s correlation test. One-way analysis of variance was used to calculate the differences in candidate reference genes between the patients and controls. Statistical analyses were performed using GraphPad Prism (GraphPad Software, Inc., San Diego, CA, USA). All statistical tests were two-sided, and a *p*-value < 0.05 was considered statistically significant. The *p*-values were corrected for multiple testing using Bonferroni correction.

## 3. Results

### 3.1. Demographic and Clinical Features of Patients

Twenty-six patients were included in the analysis, and 69% of the patients were male with a mean age at lung transplantation of 48.5 ± 10.5 years. The most represented underlying diagnosis was chronic obstructive pulmonary disease (COPD, 27%), idiopathic pulmonary fibrosis (IPF, 23%) and cystic fibrosis (CF, 15%). The preferred type of transplantation was bilateral (69%). After BOS diagnosis, formulated according to recent ISHLT consensus documents [28], sixteen patients were treated by on-line and ten patients by off-line ECP, respectively. After 5.9 ± 1.7 months of treatments and 17.5 ± 6.2 procedures, patients’ clinical and functional responses were evaluated. Serum samples were collected from all enrolled patients at the timeline defined above. Peripheral blood mononuclear cells (PBMCs) and BAL supernatants were collected at both time points in a subsample of enrolled patients (*n* = 4). All patients were treated with calcineurin inhibitors (Tacrolimus or Cyclosporin A) in combination with low-dose steroids; in addition, most patients also received Mycofeolate Mofetil. All patients had experienced a 3 month-course of low-dose Azithromycin before starting ECP. There was no difference between those patients that were treated by off-line or on-line ECP in terms of ECP outcome at the last follow-up. According to the stabilization or improvement of lung function from basal ECP entry value, 8 (30%) patients were classified as non-responders, while the remaining 18 (70%) patients were judged as responders after 6 months of ECP treatment. Table 1 summarizes patient population features.

### 3.2. Serum Expression Levels of Selected miRNAs

**Figure 1:**
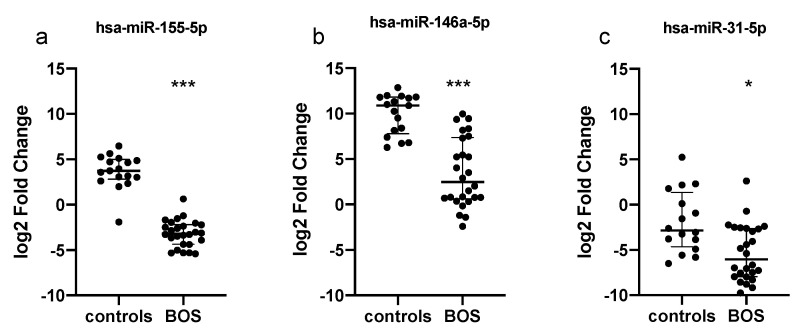
Quantitative expression of (**a**) miR-155, (**b**) miR-146a and (**c**) miR-31 in serum samples from 26 patients before ECP therapy and 17 healthy subjects. Relative expressions were expressed as log2 transformed values. *** *p* < 0.0001; * *p* < 0.05.

Among the analyzed miRNAs, we observed a significant downregulation of hsa-miR-155-5p, hsa-miR-146a-5p (both Bonferroni adjusted *p*-value < 0.0001) and hsa-miR-31-5p (adjusted *p*-value = 0.015) in LTx patients at ECP entry when compared to healthy controls (Figure 1). In Supplementary Table S1, we reported the overall results of the 14 miRNAs in the studied population.

**Figure 2:**
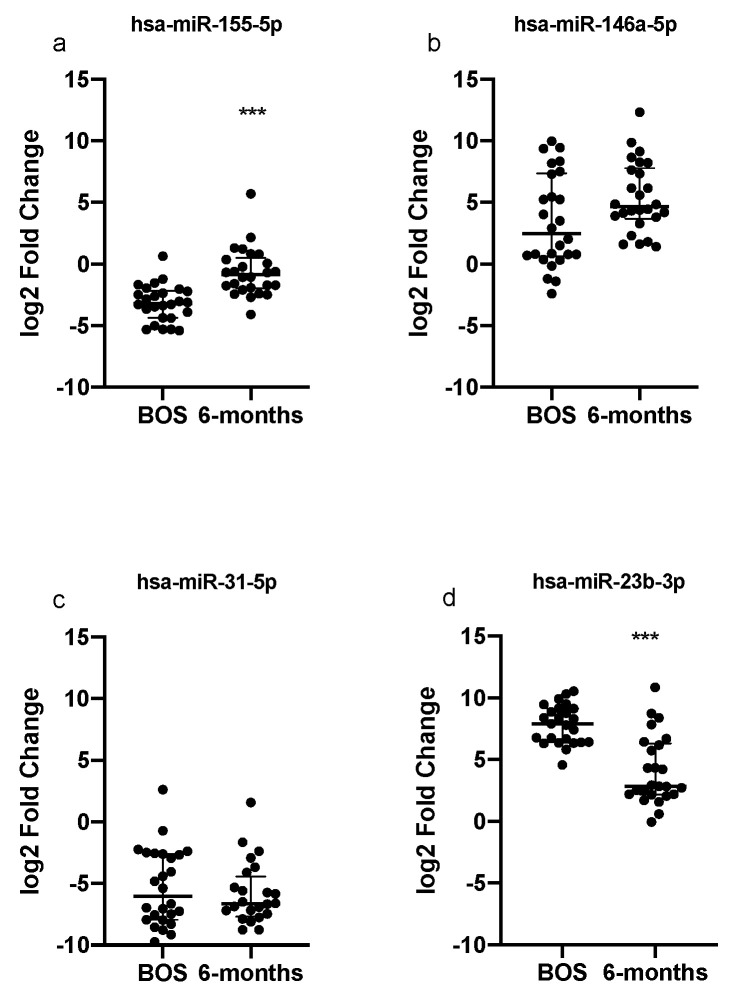
Quantitative expression of (**a**) miR-155, (**b**) miR-146a, (**c**) miR-31 and (**d**) miR-23 in serum of patients before ECP therapy and after 6 months of treatment. Relative expressions were expressed as log2 transformed values. *** *p* < 0.0001.

After 6 months of ECP therapy, paired analysis showed hsa-miR-155-5p expression levels increased with respect to the pre-ECP levels (Bonferroni adjusted *p*-value < 0.0001); however, they did not reach the levels observed in the healthy population sera. Conversely, hsa-miR-146a-5p and hsa-miR-31-5p expression levels did not significantly change after 6 months ECP. ECP treatment also induced a significant reduction in hsa-miR-23b-3p expression levels (Bonferroni adjusted *p*-value < 0.0001), which were comparable to healthy controls at start of ECP (Figure 2).

**Figure 3:**
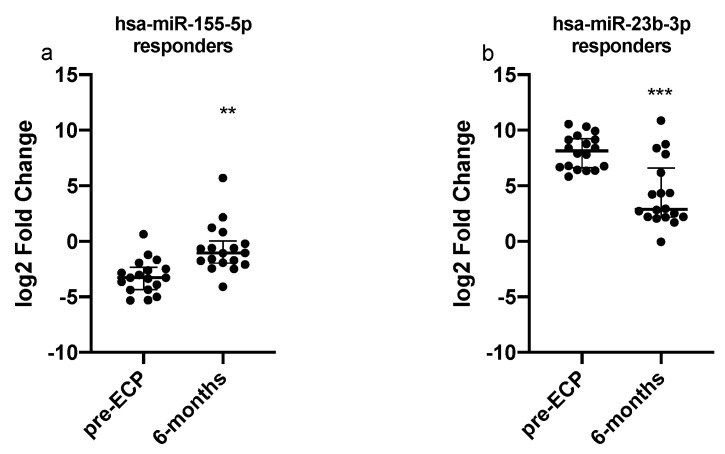
Quantitative expression of (**a**) miR-155 and (**b**) miR-23b assessed by qRT-PCR in responder patients. Relative expressions were expressed as log2 transformed values. *** *p* < 0.001; ** *p* < 0.01.

In order to investigate whether any miRNAs could predict the response to ECP, we compared miRNAs baseline levels in responders and non-responders; however, we could not detect any significant difference between these two groups of patients. With the aim of evaluating the role of miRNAs in the molecular mechanisms underlying the response to ECP, we assessed their levels in patients who actually showed a clinical response to therapy. By limiting the analysis to responders, a significant increase in hsa-miR-155-5p (*p* = 0.0056) and a significant downregulation of hsa-miR-23b-3p in serum (*p* < 0.001, Figure 3) was observed.

### 3.3. Signaling Pathway Prediction and Targets Analyses

A DIANA-mirPath analysis was applied to predict the biologic targets and pathways, as well as cellular processes, modulated by the dysregulated miRNAs (hsa-miR-155-5p and hsa-miR-23b-3p). The following signaling pathways were found to be enriched: TGF-beta signaling pathway, steroid biosynthesis, adherens junction, hippo signaling pathway, FoxO signaling pathway, chronic myeloid leukemia, ECM–receptor interactions, hepatitis B, apoptosis, non-small cell lung cancer, proteoglycans in cancer, signaling pathways regulating pluripotency of stem cells, colorectal cancer, pathways in cancer, endometrial cancer and bladder cancer (Table S2). Some of the identified pathways were involved in the processes of lung fibrogenesis and graft tolerance [24,25,26,27]. We decide to concentrate further on three targets reported as deeply involved in these processes: SMAD3, SMAD4 and STAT3.

### 3.4. Dysregulated miRNAs and Selected Targets Expression Analyses in PBMCs

**Figure 4:**
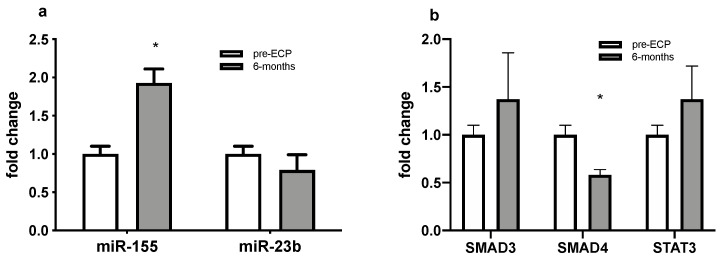
Quantitative expression of (**a**) miR-155 and miR-23b and (**b**) SMAD3, SMAD4 and STAT3 assessed by qRT-PCR in PBMCs of BOS patients. * *p* < 0.05.

In order to evaluate the possible relations between miR-155-5p and miR-23b-3p expression levels and SMAD3, SMAD4, STAT3 mRNA levels, RNA isolated from PBMCs were purified at time of ECP initiation and after 6 months of ECP treatment in four patients of the included sample. A paired analysis showed increased expression levels of hsa-miR-155-5p in PBMCs with respect to the pre-ECP levels (*p* = 0.016; Figure 4a). Conversely, hsa-miR-23b-3p showed no significant differences after 6 months ECP from baseline. The upregulation of miR-155-5p was not associated with a significant dysregulation of SMAD3 and STAT3 mRNA levels in PBMCs, while a significant downregulation of SMAD4 mRNA levels was detectable (*p* = 0.027; Figure 4b).

### 3.5. Expression Levels of Dysregulated miRNAs and TGF-b Levels in BAL Supernatant

**Figure 5:**
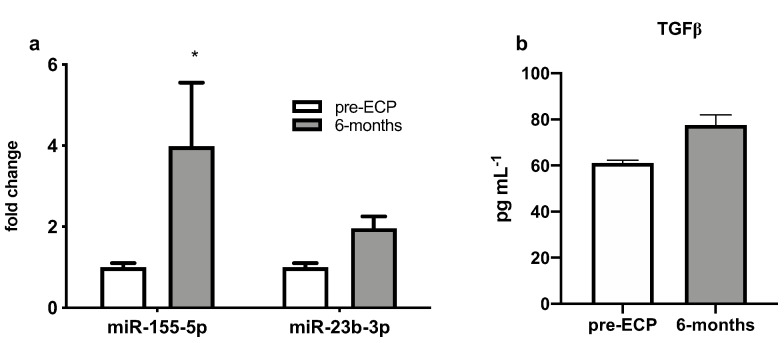
(**a**) Quantitative expression of miR-155 and miR-23b assessed by qRT-PCR and (**b**) TGFβ levels by ELISA in BAL supernatant of BOS patients. * *p* < 0.05.

After 6 months of ECP therapy, a paired analysis showed that expression levels of hsa-miR-155-5p increased with respect to the pre-ECP levels (*p* = 0.02) in BAL supernatant of BOS patients, mirroring what was observed for the same miRNA in serum samples. No significant differences were observed in hsa-miR-23b-3p expression levels (*p* = 0.41), even though its circulating levels were decreased following the treatment (Figure 5a). After 6 months of treatment we observed a non-significant trend towards an increase in TGF-beta BAL fluid levels in BAL supernatant the TGF-β1 levels with respect to basal values (Figure 5b).

## 4. Discussion

ECP therapy has been reported to reduce the decline of respiratory function and improve survival in BOS patients in several retrospective monocentric reports [29,30,31]. The technique adopted to perform the procedure (inline or off-line) does not seem to influence the clinical response to the treatment. Nonetheless, some differences exist regarding the patient’s management and the product collected [32,33]. A paired trial compared mononuclear cell collection in two machines for the further inactivation through an inline or offline extracorporeal photopheresis procedure [34]. The comparison of procedure times and collection efficiencies using integrated and multistep nonintegrated procedures for extracorporeal photopheresis [35]. The off-line method can easily be tailored to the patient’s characteristics and offers the advantage of a low extracorporeal volume of the cell separator device (140–195 mL) with a positive impact on fluid balance, which can also treat patients with haemoglobin levels until 7 gr/dL with no transfusion support. Furthermore, there is an excellent flexibility for managing the patients’ venous accesses, often avoiding the central venous catether positioning. Furthermore, the mononuclear cell yield and purity are higher than those obtained with the on-line system, even if no differences in terms of clinical results and the amount of mononuclear cells infused are reported to date. On the other hand, an apparent advantage of the on-line system consists of avoiding the cell product handling in the laboratory and the related risks.

Different studies evaluated the immunomodulatory effect of ECP therapy on patients after solid organ transplantation [36,37,38,39], but few studies have explored the molecular regulation associated with ECP treatment. Although the exact mechanism how ECP modulates the immune system are not fully understood, previous works on murine model and on pediatric transplant recipients [40,41] have suggested that ECP induces expansion of Treg and/or tolerogenic DCs. Moreover, it was demonstrated that ECP might influence the frequency of circulating Tregs [7]. Treg promotes a state of antigen-specific peripheral tolerance by suppressing the activation and expansion of reactive effector cells [42]. Likewise, Tregs number and function might also be excellent biomarkers of allograft injury and function, and the balance of Thelper/Treg can offer insights into allograft rejection [43]. The analysis of variations in miRNAs peripheral levels might, therefore, be of help in clarifying molecular mechanisms of ECP. Furthermore, aside from the mechanistic utility, a specific miRNA signature could be of help as an easily accessible biomarker to predict ECP response [44,45].

To date, no studies have addressed the variation in miRNAs expression during ECP treatment in patients with BOS. Only one previous report was focused on patients with graft-versus-host disease (GVHD) and showed that GVHD patients initiating ECP had higher miR-22-5p, miR-34a-5p, miR-148a-3p, miR-505-3p in comparison to healthy controls, and those patients who responded to treatment normalized the levels of these micro-RNAs after 6 months of ECP. However, in the same study, the role of the above miRNAs in response to ECP in BOS patients was not confirmed [46].

In our study, we found a different expression profile of specific circulating immunoregulatory miRNAs. In fact, in patients with BOS, at time of ECP enrolment miR-155-5p, miR-146a-5p and miR-31-5p were significantly downregulated with respect to healthy controls. Of note, miR-155-5p is a key player in the regulation of adaptive immunity and antibody-related T-cell response [47]. Local inflammation associated with rejection is tightly regulated by T helper (Th)/Treg balance, and miR-155 controls the differentiation of CD4+T cells into Th cells and participates in the development of regulatory T (Treg) cells [22,48,49]. It has been proposed that several miRNAs are direct targets of FOXP3, such as miR-155, and may partly control the function, development and homeostasis of Tregs [50]. Similar to miR-155, miR-146a expression was elevated in Tregs and it was induced upon activation [50]. MiR-146a deficiency results in the increased production of pro-inflammatory interferon-gamma (IFNγ), altering their function and ability to preserve immunological tolerance. The significant downregulation of miR-155 and mir-146a in patients experiencing BOS in our cohort is in accordance with the above observations, thus suggesting that the levels of these miRNAs might mirror the level of immunological tolerance of these subjects. However, due to the limited sample size of our study, data need to be confirmed in a larger population of CLAD patients, in order to accurately assess the utility of miR-155 and mir-146a as disease biomarkers.

Furthermore, we observed that ECP was able to induce a significant increase in circulating levels of miR-155 after 6 months, even if it did not induce restoration to levels of healthy controls. A previous study, conducted in liver transplant patients, showed an association between miR-155 and clinical operational tolerance, with higher miR-155 circulating levels in patients without rejection episodes for at least one year [51]. In our patients, the upregulation of miR-155 was also present in BAL supernatant after 6 months of treatment.

Another miRNA that was significantly downregulated in BOS patients at time of ECP initiation with respect to healthy controls was miR-31. However, in these patients, ECP was not able to substantially affect its level. Several studies demonstrate that the function of miR-31 is context-dependent. As part of the immune response, miR-31 regulates Treg cells through several mechanisms [51]. Furthermore, recent literature points out that miR-31 is also a negative regulator of fibrogenesis and pulmonary fibrosis [52]. Nevertheless, miR-31 does not seem to be linked to the molecular mechanisms of ECP clinical response.

Unexpectedly, although miR-23b levels did not differ between controls and patients at baseline (pre-ECP), we observed that this miRNA was significantly downregulated with ECP treatment. Previous data in the literature showed conflicting results with respect to immune regulation. On the one hand, miR-23b was defined as a pro-tolerogenic factor, increasing tolerogenic DC activity and Treg responses in vitro through the inhibition of the Notch1 and NF-κB signaling pathways [53]. On the other hand, in the context of autoimmune disorders, specifically rheumatoid arthritis [54], levels of miR-23b expression were directly correlated to disease activity [55]. In addition, miR-23b was ascribed a pathogenic role in the context of cardiac fibrosis. Thus, aside from an immune-regulatory role, a possible pro-fibrogenic role could also be speculated in miR-23b in the context of obstructive CLAD, and this needs to be fully clarified.

Finally, by the analysis of the different pathways in KEGG database, some common targets for miR-155-5p and miR-23b-3p were identified. Surprisingly, these pathways and targets were also involved in fibrogenesis, in particular in TGF-β-driven processes. Thus, with the aim to analyze whether miR-155 and miR-23-b deregulation might be associated with an increased expression of some crucial targets in fibrogenic processes, we specifically analyzed the mRNA expressions of STAT3, SMAD3 and SMAD4 in the PBMCs of a subset of patients undergoing ECP, and we compared their expression at 6 months post-ECP with respect to basal time point. Neither STAT3 nor SMAD3 showed a variation related to ECP treatment, while SMAD4 was significantly downregulated at 6 months with respect to the basal time point. At same time points, an upregulation of miR-155-5p was also detectable in PBMCs. This finding was in line with the observation obtained for serum samples, thus suggesting that SMAD4 expression was regulated by miR-155-5p at the transcriptional levels, this miRNA a repressor of SMAD4 mRNA. The functional role of SMAD4 in the pathogenesis of inflammation and fibrosis under pathological condition remain largely unknown. SMAD4 expression was shown to regulate LPS tolerance through the regulation of SHIP1 and IL-1R-associated kinase (IRAK)-M, negative regulators of TLR4 signaling [27]. In addition, the central role of SMAD4 in TGFβ signaling pathway is well known [56]. Recent studies in conditional SMAD4 knockout mice have shown that SMAD4 may be a key regulator for different roles of TGFβ in immunoregulation and fibrogenesis by interacting with SMAD7 and SMAD3 [57]. In addition, SMAD 4 downregulation is able to inhibit renal fibrosis [58,59]. Indeed, in BAL samples, we could not detect a significant variation of TGF-beta levels after ECP treatment, but only a trend towards an increase. Further mechanistic studies are therefore needed to clarify the regulation of TGF-beta pathway by ECP.

We acknowledge that our study has some limitations. First, the quantification of different immune cell subsets is lacking. This depends on the availability of the biological material of the patients enrolled in the study, including the samples of PBMCs and BAL supernatants. Second, the number of non-responder patients in our case series was small. However, our pilot study represents the first study describing different miRNAs expression in BOS patients treated with ECP and may suggest a possible underlying process.

## 5. Conclusions

In conclusion, from our study, a specific miRNA signature associated with a functional stabilization/improvement after an ECP treatment course of 6 months emerged. The kinetic of miR-155 in particular, and of miR-23b, might be useful for identifying patients who are responsive to ECP treatment. These results also suggest that ECP might not only induce immune tolerance, as previously suggested, but also possibly interfere with lung fibrogenic pathways by means of miR-155 and miR-23b modulation. These data provide a theoretical basis for subsequent longitudinal clinical and mechanistic studies, including target gene determination and function analysis.

## Figures and Tables

**Table 1:** Demographic and clinical data of study subjects.

	On-Line (*n* = 16)	Off-Line (*n* = 10)
Males (*n*, %)	11 (69%)	7 (70%)
Age at transplant (mean, DS)	49 ± 11	43 ± 7
Age at ECP (mean, DS)	54 ± 11	52 ± 9
Underlying diagnosis: Chronic obstructive pulmonary disease (COPD) Idiopathic pulmonary fibrosis (IPF) Cystic fibrosis (CF) Other	7 (44%) 2 (12.5%) 1 (6%) 6 (37.5%)	0 4 (40%) 3 (30%) 3 (30%)
Bilateral lung transplantation (*n*, %)	11 (69%)	7 (70%)
ECP outcome at last follow-up: Responder Non responder	11 (69%) 5 (31%)	7 (70%) 3 (30%)

## Data Availability

Data related to this manuscript is contained within the article or Supplementary material.

## Supplementary Materials

The following supporting information can be downloaded at: www.mdpi.com/article/10.3390/cells11071117/s1, Table S1: Specific common targets of deregulated miRNAs and enriched pathways. Table S2: Pathways predicted by DIANA-mirPath analysis.
