# Supplementary material for: Mechanisms of Action of Extracorporeal Photopheresis in the Control of Bronchiolitis Obliterans Syndrome (BOS): Involvement of Circulating miRNAs
Source: Cells. 2022 Mar 25;11(7):1117. doi: 10.3390/cells11071117 (PMC8997705; doi:10.3390/cells11071117)
Supplement: Supplementary file 1 [file cells-11-01117-s001.zip › cells-1614412-supplementary.pdf]

Table S1: Overall results of miRNAs expression levels in studied population expressed in log2 transformed values

	Controls			pre-ECP			6-months			Adjusted *p*-value	
miRNAs	Median	Percentile 25	Percentile 75	Median	Percentile 25	Percentile 75	Median	Percentile 25	Percentile 75	BOS vs CTR	Pre-ECP vs 6-months
hsa-miR-155-5p	3.74	2.82	4.98	−3.19	−4.37	−2.18	−0.69	−1.97	0.48	<0.0001	<0.0001
hsa-miR-146a-5p	10.89	7.78	11.81	2.47	0.62	7.36	4.47	3.68	7.79	<0.0001	>0.9999
hsa-miR-31-5p	−2.84	−3.64	1.37	−6.02	−7.95	−2.61	−6.65	−7.69	−4.42	0.015	>0.9999
hsa-miR-21-5p	10.29	9.17	11.83	8.85	6.85	10.53	8.46	6.77	9.97	0.199	>0.9999
hsa-miR-223-5p	3.41	2.76	5.23	4.30	2.60	5.24	4.72	2.54	6.40	>0.9999	>0.9999
hsa-miR-125a-5p	3.54	2.81	3.97	3.37	2.08	5.79	4.96	2.94	6.48	>0.9999	>0.9999
hsa-miR-30b-5p	5.21	3.97	6.80	5.92	3.44	6.30	5.89	3.94	7.15	>0.9999	>0.9999
hsa-miR-24-3p	4.13	3.54	4.56	5.25	3.89	5.90	8.13	6.18	12.51	>0.9999	>0.9999
hsa-miR-98-5p	−0.44	−0.59	−0.13	1.72	−0.72	2.42	1.01	0.33	3.07	>0.9999	>0.9999
hsa-miR-99a-5p	−3.07	−3.80	−2.99	−2.33	−2.51	−1.80	−1.95	−2.67	−1.39	>0.9999	>0.9999
hsa-miR-23b-3p	7.68	6.50	9.09	7.91	6.41	9.14	2.85	2.17	6.31	>0.9999	<0.0001
hsa-miR-182-5p	1.84	1.15	2.22	−0.49	−1.83	1.39	−0.74	−1.48	1.33	>0.9999	>0.9999
hsa-miR-17-5p	7.55	6.88	8.04	5.32	1.64	6.47	4.37	1.82	6.00	0.635	>0.9999
hsa-miR-181a-3p	−4.36	−5.60	−2.45	−3.14	−3.75	−2.60	−3.17	−4.09	−2.94	>0.9999	>0.9999

Table S2. Pathways predicted by DIANA-mirPath analysis.

**KEGG pathway**<br>**hsa-miR-23b; hsa-miR-155**	**p-value**	**genes**
TGF-beta signaling pathway	<0.00001	THBS1<br>**SMAD3**<br>**SMAD4**<br>SMAD5<br>SP1<br>SMAD1
Steroid biosynthesis	<0.00001	DHCR24<br>CYP51A1
Adherens junction	0.0003	MET<br>CTNND1<br>**SMAD3**<br>EGFR<br>TJP1<br>**SMAD4**<br>CTNNB1<br>PTPRJ
Hippo signaling pathway	0.0007	YWHAE<br>PPP1CC<br>**SMAD3**<br>MPP5<br>CCND1<br>**SMAD4**<br>CTNNB1<br>MOB1A<br>SMAD1<br>PPP1CB
FoxO signaling pathway	0.0007	**STAT3**<br>PCK2

		**SMAD3**<br>EGFR<br>CCND1<br>**SMAD4**<br>FOXO3<br>PLK1<br>SGK3<br>MDM2
ECM-receptor interaction	0.0011	ITGB1<br>THBS1<br>COL4A2
Hepatitis B	0.0013	**STAT3**<br>**SMAD3**<br>BCL2<br>MAVS<br>MYD88<br>CCND1<br>**SMAD4**<br>E2F3
Apoptosis	0.0065	BCL2<br>MYD88<br>MAP3K14<br>TNFRSF10B<br>PRKAR1A<br>XIAP
Non-small cell lung cancer	0.0065	EML4<br>EGFR<br>CCND1<br>E2F3<br>FOXO3
Proteoglycans in cancer	0.007	**STAT3**<br>MET<br>ITGB1<br>THBS1<br>PPP1CC<br>EGFR<br>CCND1<br>CTNNB1<br>MDM2<br>PPP1CB
Signaling pathways regulating pluripotency of stem cells	0.0324	**STAT3**<br>**SMAD3**<br>**SMAD4**<br>CTNNB1<br>SMAD5<br>IL6ST<br>SMAD1
Colorectal cancer	0.0324	**SMAD3**<br>BCL2<br>CCND1<br>**SMAD4**<br>CTNNB1

Pathways in cancer	0.034	**STAT3**<br>MET<br>ITGB1<br>**SMAD3**<br>BCL2<br>EGFR<br>CCND1<br>**SMAD4**<br>CTNNB1<br>COL4A2<br>E2F3<br>MDM2<br>XIAP
Endometrial cancer	0.034	EGFR<br>CCND1<br>CTNNB1<br>FOXO3
Bladder cancer	0.0351	THBS1<br>EGFR<br>CCND1<br>E2F3<br>MDM2
